# Influence of Wastes and Synthesis Conditions on the Compressive Strength, Setting Time and Gels of Waste-Based Geopolymers

**DOI:** 10.3390/gels10110700

**Published:** 2024-10-29

**Authors:** Tianyu Wang, Feng Rao, Lang Yang, Kaixi Jiang, Nanan Lin, Liwei Mo

**Affiliations:** 1 School of Materials Science and Engineering, Fuzhou University, Fuzhou 350108, China; wangtianyu950709@163.com (T.W.); jiangkx@bgrimm.com (K.J.); 2 Zijin School of Geology and Mining, Fuzhou University, Fuzhou 350108, China; siryanglang@fzu.edu.cn (L.Y.); www15930025306@163.com (N.L.); 19959111778@163.com (L.M.); 3 Fujian Key Laboratory of Green Extraction and High-Value Utilization of New Energy Metals, Fuzhou University, Fuzhou 350108, China

**Keywords:** iron ore and blast-furnace slag residues, waste-based geopolymer, setting time range, rapid setting, gel evolution, microstructure of geopolymer, geopolymer building materials

## Abstract

In civil engineering, both rapid setting and delayed setting are needed for various application scenarios. In order to regulate the setting time of concrete, the iron ore tailings-blast furnace slag (IOT-BFS)-based geopolymers were synthesized with a broad range of setting time and a high compressive strength in this study. The factors of iron ore tailings content, alkali content, liquid–solid ratio, and modulus of alkali activator on setting time of the geopolymers were analyzed. The setting times of geopolymers are tested by a manual Vicat apparatus, and their microstructure is characterized by scanning electron microscopy (SEM), as well as that the hydration heat flow is characterized by an isothermal heat conduction calorimeter (TAM Air). It is found that setting time of the geopolymers was mostly affected by the modulus of alkaline activators due to the reasons that changes in modulus lead to the accelerated hydration reaction, formation of low-polymeric silicates, generation of gels, and encapsulation of precursor particles caused by high viscosity. Adjusting the modulus of the alkaline activator to 0.8 can control the initial setting time of the geopolymers to around 3 min. When the modulus ranges from 1–1.8, the initial setting time fell in the range of 15–45 min. For an alkaline activator modulus of 2, the initial setting time increased to 108 min. This study gives a clue for the preparation of geopolymers with adjustable setting times for multi-scenario applications in construction materials.

## 1. Introduction

In civil engineering, concrete with various setting times is needed in different application scenarios [1]. Repair of airport runways, tunnels, bridges, etc., requires rapidly setting concrete [2], while others, like underground building structures (underground pipelines) [3], buildings constructed in high-temperature environments, and long-distance transportation of ready mixed concrete demand concrete with retarded setting [4]. In several applications, such as 3D printing [5] and oil well operations [6], more precise control of the concrete setting time is required to meet specific engineering needs. During the process of oil well operations, the slurry must remain in a fluid state for several hours because it becomes difficult to pump efficiently when its viscosity exceeds a certain limit [7]. However, in order to shorten the valuable rig time, the slurry needs to solidify as quickly as possible after being pumped [8]. Concrete used in 3D printing should not set before extrusion in order to avoid clogging the printing system [9]. However, it needs to set quickly after deposition to withstand subsequent accumulated weight. Geopolymers, synthesized through activating aluminosilicate materials with alkaline solutions, exhibit comparable mechanical properties and low CO_2_ emissions compared to ordinary Portland cement (OPC) [10]. Recently, geopolymer is considered a cementitious material with the potential to replace OPC [11]. However, most research investigated the mechanical properties of geopolymers but ignored the regulation of setting time [12].

Some research studied the impact of synthesis conditions on the setting time of geopolymers [13]. Firstly, different types and particle sizes of raw materials lead to varying reaction activities [14], which affect the setting time of geopolymers [15]. Ma et al. [16] evaluated the effect of ground granulated blast furnace slag (GGBS) content on the setting time of fly ash-GGBS-based geopolymers. The difference in initial setting time between geopolymers with 50% GGBS and geopolymers with 20% GGBS can approach approximately 150 min. It can be attributed to the increase in CaO content in the paste, which results in sufficient Ca^2+^ ions for the silica–aluminous raw material to generate multiple coagulation nodules during the process of depolymerization, leading to a compact gel and a decrease in setting time. Kim et al. [17] investigated the effect of calcium on the setting behavior of metakaolin-based geopolymers. The results show that the setting time of geopolymers was accelerated in proportion to the dosages of calcium hydroxide in up to 4% of the total mix weight. The fast setting is caused by the fast dissolution of metakaolin and precipitation of C-S-H gel. Other additions like waste glass [18] and gangue powder [19] could also decrease the setting time of geopolymers because of the high activity. In addition, yellow river sediment (YRS) and red mud were used to prepare porous concrete-based geopolymer. The results show that the addition of YRS can effectively prolong the setting time of the geopolymer (150% compared with the geopolymer without YRS). This is because the low activities of the materials caused by YRS hinder the geopolymerization [20]. For geopolymers with various particle sizes, the final setting time of geopolymers with 9 μm fly ash is reduced by more than 130 min compared to geopolymers using 25 μm fly ash. It is mainly due to the fact that smaller particles have a higher amount of amorphous materials, which can be directly correlated with higher reactivity. Next to this, more surface area is in contact with the alkali activator for smaller particles, thus accelerating the dissolution of raw materials [21].

Secondly, the variation in type and properties of alkali activators can also influence the setting time of geopolymers by modifying the nature of cations and silica–alumina ratio in the alkaline solution. Li et al. synthesized the fly ash-slag-based geopolymers and investigated the effect of alkali content on setting time. The results show that the setting time increased dramatically with alkali content increasing. The reason is that the increasing Na_2_O hinders the dissolution of raw materials, resulting in a slower formation of C-S-H gel [22]. For geopolymers employing sodium silicate and sodium hydroxide as alkali activators, an increase in the ratio of sodium silicate to sodium hydroxide leads to a reduction in setting time [23]. This phenomenon can be attributed to the augmented concentration of soluble silica, which facilitates the geopolymerization [24]. In addition, a slower rate of hardening has been reported for potassium-based geopolymers compared to those of sodium [25]. The difference is mainly related to the fact that the cation with a smaller size tends to react with the small silicate oligomers, such as silicate monomers and dimers [26].

Thirdly, synthesis conditions such as heat treatment and liquid-to-solid ratio of geopolymer paste can affect the setting time by altering the progress of geopolymerization. An acceleration effect of heat treatment on the setting of geopolymer has been reported for iron-rich laterites. Calcination of laterite containing hydroxylated minerals at 500 °C accelerates the setting time up to about 93%. It can be ascribed to the fact that calcination increases the dehydroxylation of the raw material, thus enhancing its reactivity [27]. In addition, various additives have been used to accelerate or retard the setting time of geopolymers. Initial setting time of sewage sludge ash-GGBS-based geopolymer with 3% sodium gluconate (SG) can be prolonged up to 181 min because of the potent chelation of SG and metal cations [1]. Borax was also added to the metakaolin-based geopolymer to extend the setting time by increasing the water content and modifying the polycondensation reactions to form huge structural entities [28].

However, the majority of research has primarily focused on accelerating or delaying the process of consolidation, without precisely controlling the setting time to achieve rapid setting (setting time less than 10 min) or delayed setting (setting time longer than 120 min). For example, it is necessary to accurately control the setting time of slurry to maintain a fluid state before pumping and achieve the initial setting in a few minutes after pumping [8]. In addition, most research on controlling setting time primarily focuses on adding retarders or accelerators to increase or decrease the setting time, neglecting the ability of synthesis conditions to regulate the setting time. Furthermore, there is a relative lack of systematic research on how synthesis conditions impact the setting time of geopolymers. Iron ore tailings (IOTs) are a solid waste generated by the iron ore mining industry. The present work synthesized IOT-based geopolymers with sodium silicate and sodium hydroxide. Setting times of geopolymers were regulated by altering blast furnace slag (BFS) content, alkali content, ratio of liquid to solid, and alkali activator modulus. Furthermore, the mechanism of different factors affecting the setting time of geopolymers has been investigated, and the influence of various factors on the setting time of geopolymers was compared. The objective is to prepare geopolymer concrete with high compressive strength and a wide range of setting times by varying the synthesis conditions.

## 2. Results and Discussion

**Figure 1:**
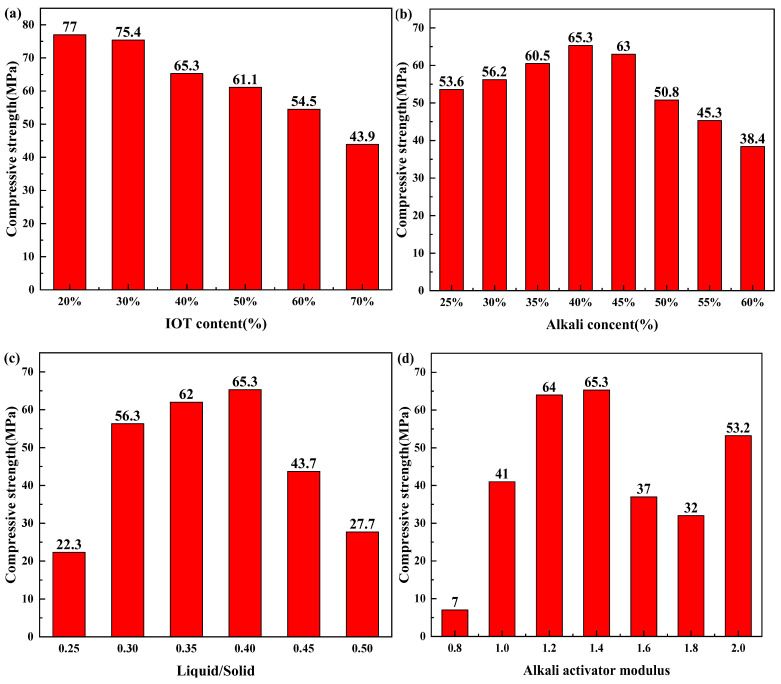
Compressive strength of IOT-BFS-based geopolymers with various synthesis conditions: (**a**) IOT content; (**b**) alkali content; (**c**) liquid/solid; (**d**) alkali activator modulus.

The compressive strength of iron ore tailings-blast furnace slag (IOT-BFS)-based geopolymers with various synthesis conditions is demonstrated in Figure 1. As the IOT content increases from 20 to 70%, the compressive strength decreases from 77 to 43.9 MPa. For geopolymers with various alkali content, compressive strength reaches the maximum value of 65.3 MPa with 40% alkali content. In addition, changes in the liquid–solid ratio also have a significant impact on the compressive strength of geopolymers. The geopolymer with a liquid–solid ratio of 0.4 has the maximum compressive strength of 65.3 MPa. For geopolymers with various moduli of alkaline activators, there exists an optimal modulus (1.4) that maximizes the compressive strength of the geopolymer.

The decline of compressive strength by increasing the IOT content can be attributed to the fact that BFS contains more active substances, such as calcium oxide, compared to IOT, which can promote the formation of geopolymer gel [29]. When the alkali content is low, partial raw materials cannot participate in the geopolymerization to form a gel, resulting in lower compressive strength [30]. When the alkali content exceeds a certain level, the self-solidification of the alkali solution also reduces the compressive strength of the geopolymer [31]. Both excessive and insufficient liquid–solid ratios can seriously affect the compressive strength of geopolymers. Low liquid–solid ratio (0.25) will cause premature consolidation of raw materials, which will stop the reaction of geopolymerization and hinder the formation of gel, thus reducing the compressive strength of geopolymer [32]. A high liquid–solid ratio (0.5) will hinder the solidification of the geopolymer and reduce its mechanical performance. A large modulus hinders the dissolution of aluminosilicate monomer, while a small modulus reduces the alkalinity of the entire system [33]. Overall, synthesis conditions have a significant influence on the compressive strength of geopolymers. Among these conditions, the modulus of the alkaline activator has the greatest impact on compressive strength. This is because changes in the modulus of the alkaline activator affect various aspects of the entire reaction system, such as changes in viscosity, variations in exothermic reactions, and the quantity of gels. The superposition of various factors causes a huge change in compressive strength. However, geopolymers synthesized under different conditions generally exhibit compressive strength exceeding 30 MPa, which meets the requirements of construction materials.

**Figure 2:**
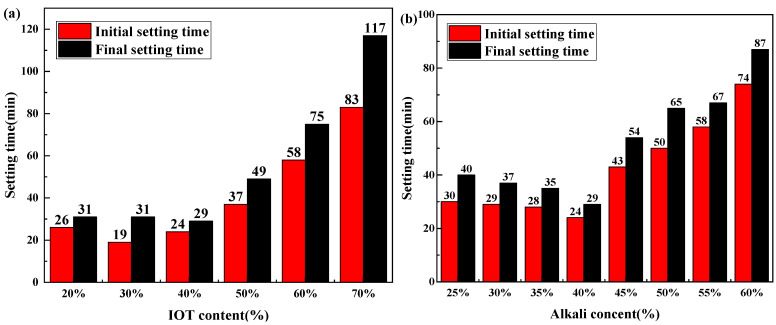

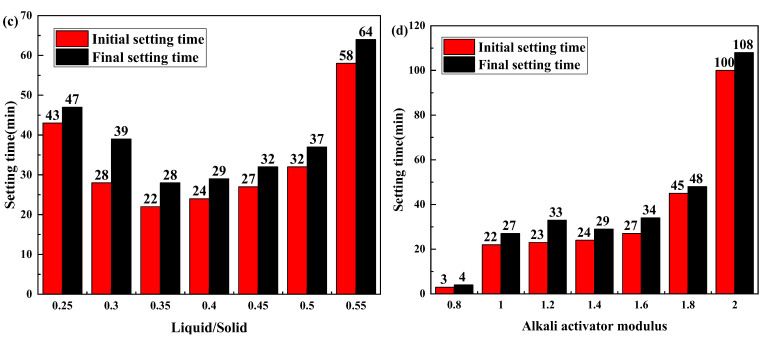
Setting time of IOT-BFS-based geopolymers with various synthesis conditions: (**a**) IOT content; (**b**) alkali content; (**c**) liquid/solid; (**d**) alkali activator modulus.

Figure 2 gives the variation in setting time for the geopolymers with different synthesis conditions. The initial and final setting times are found to first decrease and then increase with the IOT content increasing. Geopolymers containing 20, 30, and 40% IOT show similar setting times. The setting time increases instead of decreasing slightly with IOT content over 40%. Geopolymers composed of 40% IOT exhibited the fastest condensation rate, with an initial setting time of 24 min and a final setting time of 29 min. For geopolymers with various alkali content, as the initial alkali content rises, both the initial and final setting times decrease. When the alkali content exceeds a particular value, the setting time exhibits an increase as the alkali content increases. The geopolymer with 40% alkali content has the shortest initial and final setting times. A liquid–solid ratio of 0.35 or 0.4 can achieve an initial and final setting time of geopolymers within 30 min. Excessive or insufficient liquid–solid ratios will increase the setting time of geopolymers. The setting time will undergo significant changes when the modulus of the alkaline activator exceeds 1.8 or falls below 1.

It can be attributed to that high BFS content (20% IOT + 80% BFS and 30% IOT + 70% BFS) can cause part calcium to exist in the system for a free state, thereby slightly increasing the setting time of geopolymers [34]. Furthermore, IOT with less reactivity shows a low degree of geopolymerization upon blending with alkaline solution, resulting in a longer setting period [35]. When a certain amount of BFS (40% IOT + 60% BFS) was incorporated, BFS has high reactivity and a rich calcium component, which could react rapidly to form C-A-S-H gels [36]. The formation of gels consumed Ca^2+^ released from the system and improved the alkalinity within the system, thus decreasing the setting time [37]. In addition, the increasing Ca content could offer more nucleation sites for gels, leading to a shorter setting time [38]. At low alkali content (less than 40%), some raw materials cannot participate in geopolymerization to generate gel due to insufficient alkali solution. As the alkali content increases, most of the raw materials participate in the reaction, and the dissolution of calcium ions dominate. The Ca^2+^ can directly participate in the geopolymerization, which leads to a comparatively fast setting time [39]. Compared to other synthesis conditions, the liquid-to-solid has a relatively small impact on the setting time of geopolymers. It is due to the fact that the modification in the liquid–solid ratio can only alter the concentration of reactants in the system and not the amount of reactants. A high liquid–solid ratio dilutes the alkaline solution in the system, thereby increasing the setting time, while a low liquid–solid ratio affects the progress of geopolymerization and hinders the consolidation of geopolymers, thus increasing the setting time. Among these four conditions that can affect the setting time of the geopolymer, the modulus of the alkaline activator has the greatest impact on the setting time. When the modulus is greater than 1.8, silicates first react with silicates (polymerization reaction to form oligomeric silicate) and then react with the aluminate. The reaction between silicate and silicate is slow, resulting in an increase in setting time. An alkaline activator modulus less than 1 will accelerate the consolidation of geopolymer and adjust the initial and final setting time to within 10 min. Firstly, as the modulus decreases, the relative content of sodium oxide increases, which is more conducive to the dissolution of the precursor. The second is that the content of sodium hydroxide in low modulus solutions is higher, and the reaction will release more heat, which is beneficial to the progress of the geopolymerization reaction [40]. The results indicate that the setting time of geopolymers can be precisely controlled by adjusting the modulus of the alkali solution, while changing other synthesis conditions only allows the setting time to be maintained for over 20 min, failing to meet the requirement for rapid solidification.

**Figure 3:**
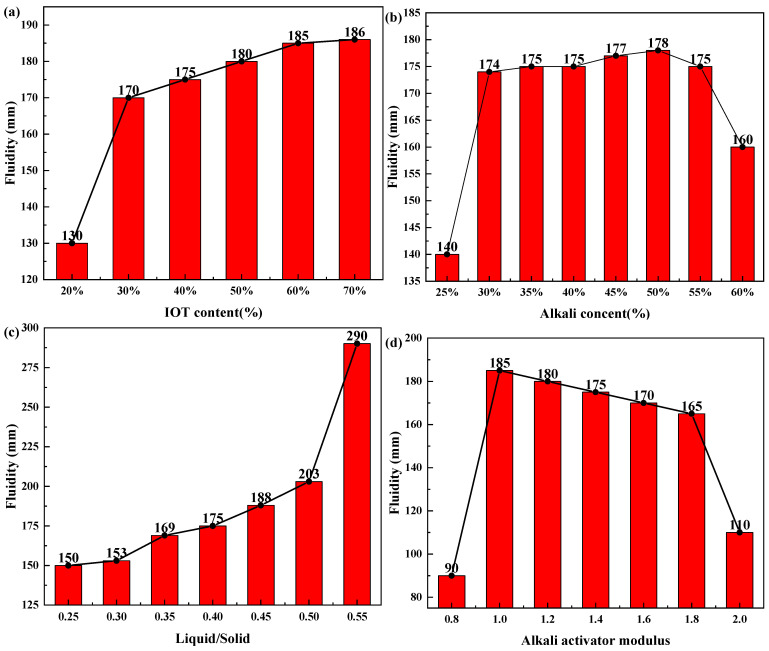
Fluidity of IOT-BFS-based geopolymers with various synthesis conditions: (**a**) IOT content; (**b**) alkali content; (**c**) liquid/solid; (**d**) alkali activator modulus.

Figure 3 presents the fluidity of the geopolymer with varying synthesis conditions. The fluidity of IOT-BFS-based geopolymers increases with increasing IOT content. At low dosing levels, the fluidity increases from 130 to 170 mm as the IOT content increases from 20 to 30%, and the change in fluidity is the greatest at this stage. For geopolymer with different alkali content, the fluidity exhibits a trend of first increasing and then decreasing as the alkali content increases. The flow diameters of the geopolymer paste with 25% and 30% alkali content are 140 and 174 mm, while with 55% and 60% alkali content, the flow diameters are 175 and 160 mm. Furthermore, liquid/solid also has a significant impact on the fluidity of geopolymer paste. The flow diameters of the geopolymer paste with a liquid–solid ratio of 0.25 and 0.55 are 150 and 290 mm. The change in fluidity caused by the variation in alkali activator modulus follows a similar trend as the change in alkali content, which also leads to an initial increase and then decrease in fluidity. The flow diameters of the geopolymer paste with 0.8 and 1.0 alkali activator moduli are 90 and 185 mm, while with 1.8 and 2.0 alkali activator moduli, the flow diameters are 165 and 110 mm.

The variation in fluidity is primarily attributed to the fact that IOT with low reactivity dispersed in the system hinders the reaction of BFS with alkaline solution, reducing the formation of geopolymer mortar. In addition, the particle size of IOT is comparatively large, and geopolymerization proceeds slowly at ambient temperature after mixing with an alkali activator, producing a geopolymer paste with high fluidity. After the addition exceeds 30%, IOT gradually distributes evenly in the system, making the fluidity steadily increase [41]. For geopolymers with various alkali content, the increase in fluidity is mainly caused by the hindrance of C-A-S-H gel formation due to the increase in Na/Al. The decrease in fluidity under high alkali content is probably related to the solidification of alkali solutions. As the liquid–solid ratio increases, the alkali solution is diluted, and the reaction proceeds more slowly. At the same time, the viscosity of the geopolymer paste decreases, leading to an increase in fluidity. The overall low fluidity of geopolymers with various alkali activator moduli is caused by high viscosity. Compared to other conditions, the liquid-to-solid ratio has the greatest impact on the fluidity of the geopolymer. This is because the fluidity of water is significantly greater than that of geopolymer paste, and an excessive or insufficient amount of water greatly affects the fluidity of the geopolymer.

**Figure 4:**
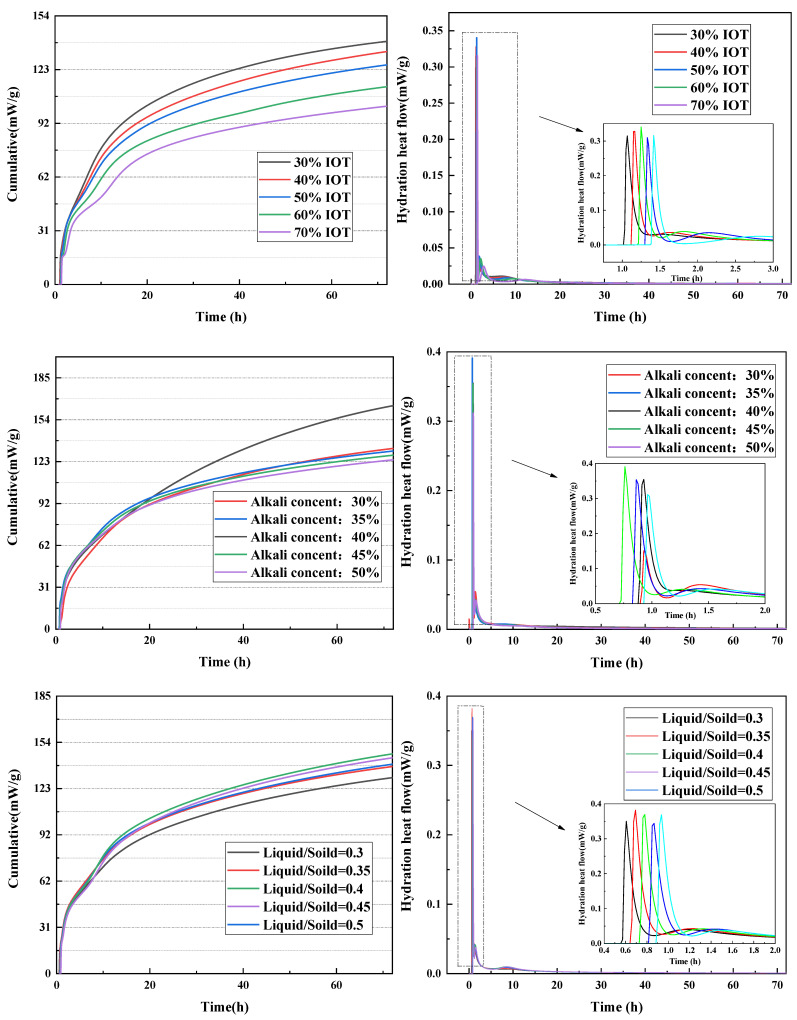

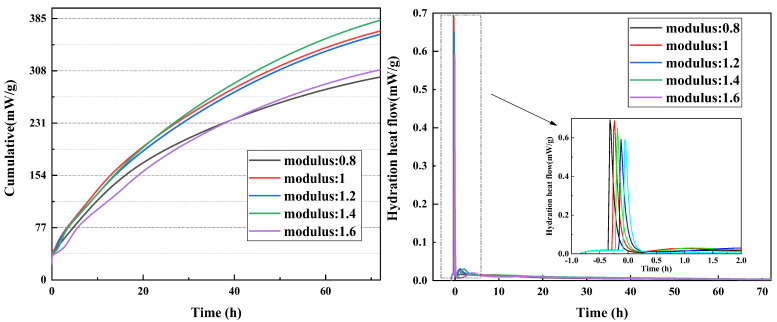
Heat flow of IOT-BFS-based geopolymer with various synthesis conditions.

Figure 4 gives the hydration heat flow and cumulative heat of IOT-BFS-based geopolymers with different synthesis conditions. The cumulative heat gradually decreases with the increase in IOT content, indicating insufficient geopolymerization and less gel formation. It can be attributed to the fact that the reactivity of BFS is much higher than that of IOT, and BFS could react with an alkaline solution to generate high compressive strength C-A-S-H gel. From the heat flow of the reaction, it can also be observed that the exothermic peak of geopolymer with 30 and 40% IOT content appears earlier than that of geopolymer with high IOT content (50, 60, and 70%), which can also lead to a reduction in setting time. For various alkali content, geopolymers with 40% alkali content exhibit the highest cumulative heat, which is also the reason for high compressive strength compared to geopolymers with other alkali content. Although the appearance of the first exothermic peak is later, the appearance of the second exothermic peak occurs earlier than that of other conditions. Moreover, excessive or insufficient liquid–solid ratio and modulus of alkaline activator can both lead to incomplete geopolymerization and low cumulative heat. There exists an optimal synthesis condition to maximize cumulative heat. However, the difference is that when the modulus of the alkaline activator is 0.8, the setting time can reach 10 min, which may be related to the earliest exothermic peak and the self-consolidation of the alkaline solution [42].

**Figure 5:**
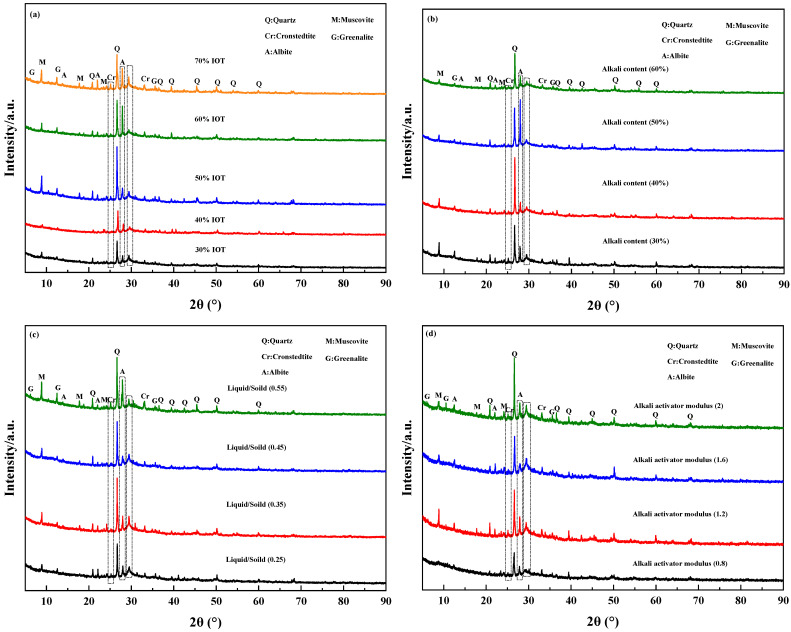
XRD spectra of IOT-BFS-based geopolymers with various synthesis conditions: (**a**) IOT content; (**b**) alkali content; (**c**) liquid/solid; (**d**) alkali activator modulus.

The X-ray diffraction (XRD) spectra of IOT-BFS-based geopolymer synthesized with different conditions are presented in Figure 5. The main crystalline phases consist of quartz, muscovite, cronstedtite, greenalite, and albite, with the hump located approximately at 27–29° 2θ, indicating the geopolymer gel [43]. The addition of IOT affects the sensitivity of gel peaks at 27–29° 2θ. The sensitivity is slightly weakened with the increasing IOT content, representing the reduction in gel. Of these, geopolymer with 40% IOT exhibits the widest peak, which corresponds to a short setting time and high compressive strength. In addition, the sensitivity of the peaks at around 25° 2θ is enhanced with the increasing amount of IOT because of the increase in iron content. For geopolymer synthesized with different alkali content, the overall crystalline phase peaks are similar, but the peak sensitivity of albite for the geopolymer with 40% alkali content decreases most significantly. This indicates a more thorough reaction, resulting in a high compressive strength and a short setting time. Additionally, the high liquid–solid ratio (0.55) will also affect the formation of the gel phase, which will produce more crystalline phases (albite, greenalite, cronstedtite, and muscovite) than other conditions. It can be inferred that when the liquid–solid ratio is between 0.35 and 0.45, the geopolymer produces more gel phase and less crystalline phase, resulting in high mechanical strength and short setting time. Compared with other conditions, geopolymer with an alkali activator modulus of 0.8 exhibits the narrowest gel peak. It is due to that the high Na^+^ ion content in the system leads to a rapid reaction, causing some raw materials to solidify before reacting. Although the setting time is short, the gel content in the system is low, and the compressive strength of the geopolymer decreases [44].

**Figure 6:**
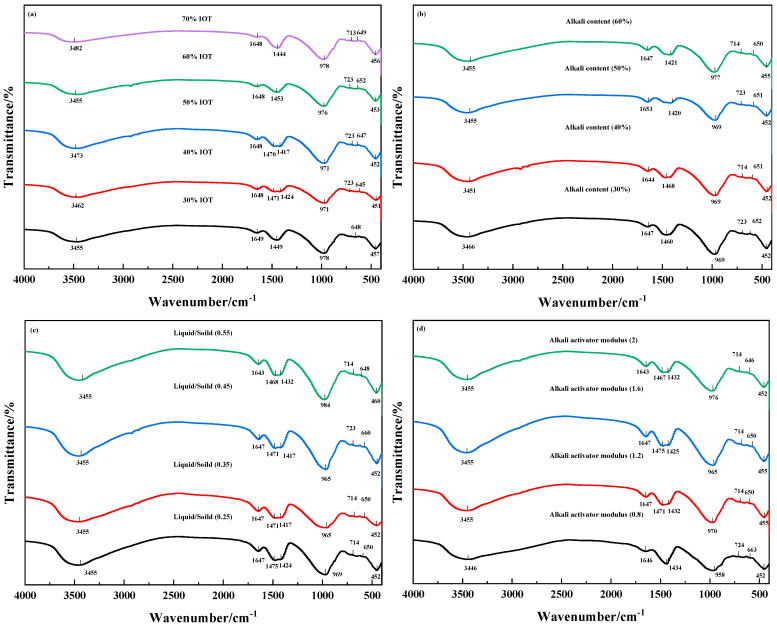
FTIR spectra of IOT-BFS-based geopolymers with various synthesis conditions: (**a**) IOT content; (**b**) alkali content; (**c**) liquid/solid; (**d**) alkali activator modulus.

Figure 6 depicts the Fourier transform infrared spectroscopy (FTIR) of IOT-BFS-based geopolymers with various synthesis conditions. The peaks at 3446–3482 cm^−1^, 1644–1648 cm^−1^ represent the stretching vibrations of OH^−^, H–OH bonds in free water [45]. The absorption peaks at 1417–1468 cm^−1^, 958–984 cm^−1^, 870–890 cm^−1^ and 714–723 cm^−1^ correspond to the asymmetric stretching of O–C–O bonds in CO_3_^2−^ groups due to carbonation during geopolymerization, the asymmetric stretching of Si–O–T in N-A-S-H gel, and the C-A-S-H gel, respectively [46]. The spectra indicate the formation of N-A-S-H and C-A-S-H gel during the geopolymer curing process.

The changes in IOT content cause the peaks around 451 and 978 cm^−1^ to shift towards the low wavenumber, representing a gradual decrease in the presence of geopolymer gel, which corresponds to a decrease in compressive strength and an increase in setting time. As the alkaline content increases, the sensitivity of the peak around 969 cm^−1^ initially increases and then decreases, corresponding to the change in Si–O–T. The results indicate the existence of an optimal amount of alkaline addition, which makes the gel structure dense and complete. However, changing the alkali content does not significantly alter the peak around 452 cm^−1^, indicating that the effect on the zeolite structure in the geopolymer is not significant [47]. For geopolymer with various liquid–solid ratios, a higher liquid–solid ratio will reduce the sensitivity of the peak around 452 and 965 cm^−1^, and the peak around 714 cm^−1^ shifts to the left, affecting the formation of C-A-S-H and N-A-S-H gel. Under various synthesis conditions, the modulus of the alkaline activator has the most significant impact on the infrared spectrum of the geopolymer. The low modulus (0.8) geopolymer only has one absorption peak around 1434 cm^−1^, which is also the effect of fast consolidation rate. The sensitivity of the absorption peak at 958 cm^−1^ is also lower than other experimental conditions, indicating that although the solidification is fast under this condition, the reaction is not sufficient, resulting in less gel and poorer mechanical performance.

**Figure 7:**
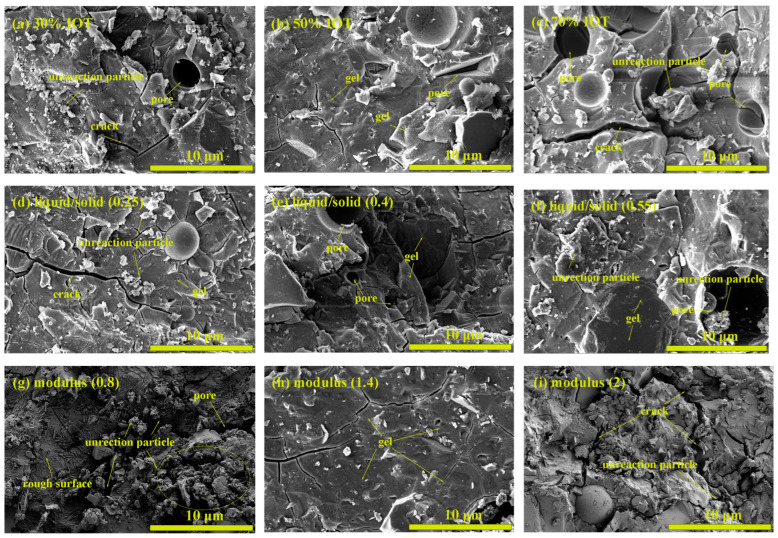
SEM images of IOT-BFS-based geopolymers at various synthesis conditions.

The scanning electron microscopy (SEM) image in Figure 7 shows the IOT-BFS-based geopolymer with various synthesis conditions. In comparison to the geopolymer containing 30% IOT, the geopolymer containing 50% IOT has a denser and smoother gel structure with fewer unreacted particles, corresponding to a thorough geopolymerization and short setting time [48]. Because of the high BFS content, there are plenty of unreacted particles in geopolymer with 30% IOT, but the geopolymer has relatively high compressive strength due to the formation of C-A-S-H gel. When the content of IOT increases to 70%, a large number of pores and unreacted particles are observed, which is due to the incomplete reaction caused by the low reactivity of IOT. The specific manifestation at the macro level is low compressive strength and long setting time. For geopolymers with different liquid-to-solid ratios, a liquid-to-solid ratio of 0.25 will cause a large number of cracks to appear on the surface of the geopolymer, reducing its compressive strength [49]. Compared to geopolymer with a liquid-to-solid ratio of 0.4, the setting time will actually increase. At a liquid-to-solid ratio of 0.55, the gel is relatively smooth, but the concentration of the alkaline solution is insufficient; some particles still cannot participate in the reaction, leading to an increase in setting time.

In addition, the modulus of the alkaline activator also has a certain influence on the microstructure of the geopolymer. When the modulus is 0.8, the gel structure of the geopolymer is relatively small, with a rough surface and a large number of cracks and unreacted particles, which is caused by an excessive amount of sodium hydroxide leading to rapid reaction. Some raw materials do not participate in the reaction, and some alkali solutions experienced self-setting phenomena. Among different alkali activator moduli, a modulus around 1.4 can produce plenty of gels and high compressive strength. However, the alkali activator modulus should be reduced to 1 if the setting time of geopolymers needs to be controlled below 10 min.

**Figure 8:**
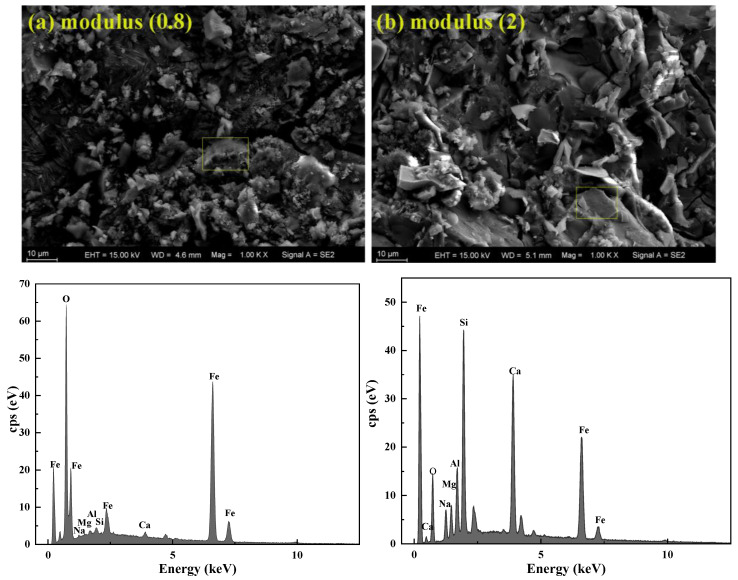
SEM images with EDS elemental mapping of Fe, Mg, Si, Ca, Al, O, and Na of IOT-BFS-based geopolymer with various modulus: (**a**) 0.8 modulus (**b**) 2 modulus.

Figure 8 shows the SEM image with energy dispersive spectroscopy (EDS) elemental mapping of Si, Ca, O, Al, Na, Mg, and Fe of IOT-BFS-based geopolymers with various alkali activator moduli. The results indicate that in the geopolymer with a modulus of 0.8, there is 49.19% Fe and 48.65% O, while only 0.55% Si, 0.44% Al, and 0.57% Ca, leading to a low content of C-A-S-H and N-A-S-H gels [50]. It is consistent with the analysis of compressive strength and setting time, confirming that a portion of the alkali solution undergoes self-setting. In the geopolymer with a modulus of 2, the content of Na, Ca, Si, and Al is 4.92%, 15.99%, 16.27%, and 5.76%, respectively, indicating a high content of gel in the geopolymer.

## 3. Conclusions

In this work, various synthesis conditions (IOT content, alkali content, liquid–solid ratio, and modulus of alkali activator) on the setting time of IOT-BFS-based geopolymers are investigated. The conclusions are as follows:

Sodium silicate and sodium hydroxide are used as alkali activators. The IOT-BFS-based geopolymer exhibits a compressive strength of 77 MPa after 7 days of curing at the synthesis conditions of 20% IOT, 40% alkali content, liquid/solid of 0.4, and alkali activator modulus of 1.4. The high compressive strength is attributed to the presence of C-A-S-H and N-A-S-H gels. After altering synthesis conditions, the compressive strength of geopolymers gradually decreases, but most of them remain above 30 MPa.

Changing the synthesis conditions of geopolymers can effectively adjust the setting time of geopolymers to meet the requirements of different applications. Among these conditions, the content of IOT and the alkali activator modulus have a significant impact on the setting time, while the alkali content and liquid-to-solid ratio have a minor effect on the setting time. All four conditions can adjust the setting time of geopolymers to be above 60 min and between 10 and 45 min. However, only when the alkali activator modulus is less than 1, the setting time can be adjusted to within 10 min. When changing the modulus of the alkaline activator, although there is some reduction in gel content, the large amount of heat released during the reaction also shortens the setting time of the geopolymer. The study has certain application prospects in the preparation of rapid-setting concrete, such as in the construction of military and civilian rapid-setting buildings, as well as in the preparation of delayed-setting concrete for applications like tunnel repair.

## 4. Materials and Methods

### 4.1. Materials

**Figure 9:**
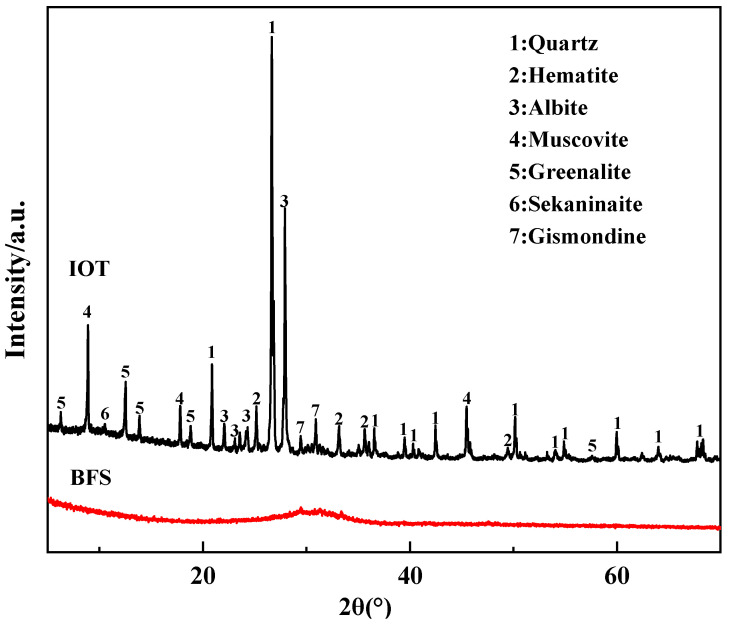
XRD patterns of IOT and BFS.

Iron ore tailings (IOT) obtained from Yunnan Province (China) and blast furnace slag (BFS) purchased from Heibei Jing Ye Steel Mill (Shijiazhuang, China) were used to prepare geopolymers. The average particle size (d_50_) was 5.2 and 7.2 μm. Sodium silicate and sodium hydroxide were collected from Shandong Yousuo Chemical Technology Co., Ltd. (Heze, China). The chemical compositions of IOT and BFS determined by X-ray fluorescence (XRF, PANalytical B.V., Almelo, The Netherlands) are listed in Table 1. The main oxides present in IOT were SiO_2_ (58.55%), Al_2_O_3_ (12.82%), and Fe_2_O_3_ (13.89%), while the main components of BFS were CaO (38.55%), SiO_2_ (30.57%), and Al_2_O_3_ (15.49%). Figure 9 shows the mineralogical characterizations of IOT and BFS collected from X-ray diffraction (XRD, Kiwa, Berlin, Germany). The major amorphous phases in IOT were quartz (SiO_2_), hematite (Fe_2_O_3_), albite (Al_1.02_Ca_0.02_Na_0.98_O_8_Si_2.98_), and a small amount of different crystalline phases like muscovite (KAl_2_(Si_3_Al)O_10_(OH,F)_2_), greenalite (H_8_Fe_6_O_18_Si_4_), sekaninaite (Fe_2_Al_4_Si_5_O_18_), and gismondine (Al_2_Ca_1_O_8_Si_2_). For BFS, there existed an amorphous peak between 25 and 35°, representing the process of pyrometallurgy.

### 4.2. Methods

**Figure 10:**
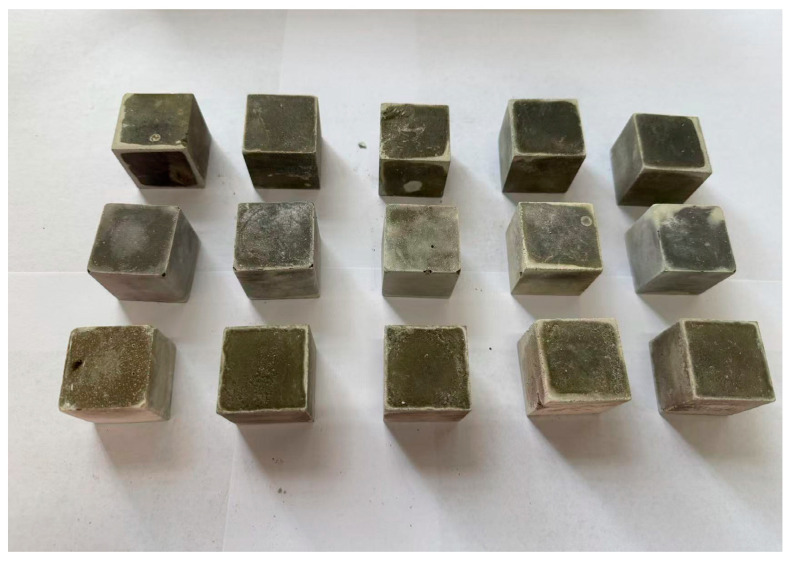
Photograph of IOT-BFS-based geopolymers.

**Figure 11:**
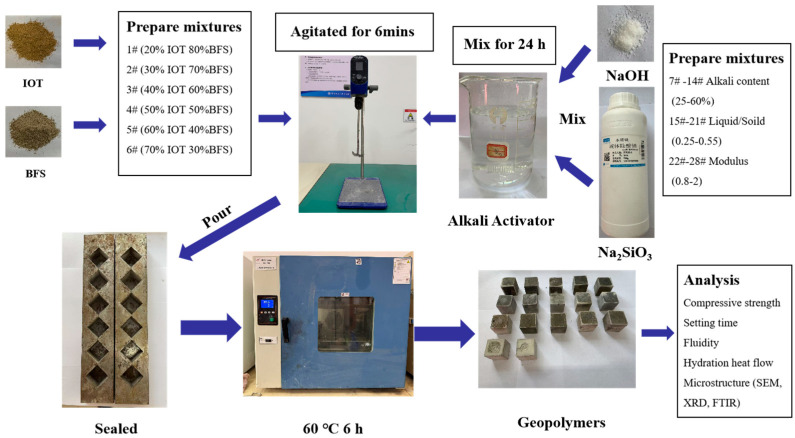
General process flow diagram for the production of IOT-BFS-based geopolymer.

Various mixes of geopolymer pastes were designed to investigate the effects of four factors on the compressive strength and setting time of geopolymers. The variables were as follows: (1) the percentage of IOT in the binder (20, 30, 40, 50, 60, and 70%); (2) the alkali content (25, 30, 35, 40, 45, 50, 55, and 60%); (3) the ratio of liquid to solid (0.25, 0.3, 0.35, 0.4, 0.45, 0.5, and 0.55); and (4) the alkali activator modulus (0.8, 1, 1.2, 1.4, 1.6, 1.8, and 2). In the synthesis of the IOT-BFS-based geopolymer, an alkali activator was prepared by mixing Na_2_SiO_3_, NaOH, and H_2_O. The blended IOT and BFS were incorporated into the alkali activator that had been left standing for 24 h and agitated for 6 min. Subsequently, the homogeneous paste was poured into steel molds (30 mm × 30 mm × 30 mm) and vibrated for 3 min to liberate entrained air. Then the molds were sealed and cured in an oven at 60 °C for 6 h and then cured at room temperature for 7 days. Table 2 gives the synthesis regime of the IOT-BFS-based geopolymer. To maintain an alkali content of 40%, a liquid-to-solid ratio of 0.35, and an alkali activator modulus of 1.4, when the raw material is 240 g, the amounts to be added were 96 g of liquid sodium silicate, 12.95 g of sodium hydroxide, and 20.64 g of water. Figure 10 shows the photographs of the IOT-BFS-based geopolymer. Figure 11 shows the general process flow diagram for the production of IOT-BFS based geopolymers.

### 4.3. Measurements

The particle size of raw materials was measured by a laser diffraction analyzer (LS-CWM, Omec, Guangzhou, China). The SEM (Quanta 250, Thermo Fisher Scientific Inc., Waltham, MA, USA) was used to observe microscopic morphology and gel structure of the geopolymers at an accelerating voltage of 0.2–30 kV. The EDS (energy dispersive X-ray spectroscopy) is used for elemental analysis of gel structure. Samples were dried and then sputtered with gold for 60 s. To obtain the mechanical properties of geopolymer, a universal testing machine (DNS100, Jinan Tiancheng Co., Ltd., Jinan, China) was used. The compressive strength result was the average value of three samples, with a variation not exceeding 10%. The microstructure of the geopolymer was obtained by using the Brucker AXS D8 X-ray diffraction meter (Shanghai Verde Instrument Technology Co., Ltd, Shanghai, China). The paste samples were first dried and then grounded to less than 75 μm to prepare specimens. CuKα_1_ radiation and a scanning rate of 0.1°/s from 5 to 90° of 2θ were used. The analysis of functional groups was completed by the FTIR spectrometer of Vector-22 (Bruker AG, Karlsruhe, Germany). The spectra were collected over a wavenumber range of 400 to 4000 cm^−1^ with a resolution of 4 cm^−1^.

The setting time of geopolymer paste was tested by a manual Vicat apparatus according to Chinese standard GB/T1346-2011 [51]. The mixed paste was poured into a truncated cone mold (65 mm × 75 mm × 40 mm), and the initial setting time was determined from the moment of adding the alkaline solution until the needle was inserted into the paste at a distance of 4 ± 1 mm from the bottom of the mold. After the initial setting time test, the mold was inverted for the final setting time test. The final setting time was recorded when the needle penetrates the paste by no more than 0.5 mm. The setting time was measured every five minutes, and after the distance between the needle and the bottom of the mold started to increase, the setting time was measured every one minute. The final result is evaluated based on three samples. The fluidity of geopolymer paste was measured according to the Chinese standard GB/T 8077-2012 [52]. The mixes were poured into a truncated cone mold (36 mm × 60 mm × 60 mm). After that, the mold was lifted to allow the slurry to naturally collapse. The fluidity was determined by measuring the maximum diameters of the two vertical directions of the slurry. Three samples were measured for the final results. The hydration heat flow of the geopolymer paste was obtained by an isothermal calorimeter, TAM Air, to estimate the effect of various synthesis conditions on the hydration process and setting time of the geopolymer. The total binder (including solid powder and alkali activator) content is 5 g. The test was conducted under a constant temperature of 20 ± 1 °C for 72 h.

## Figures and Tables

**Table 1:** Chemical compositions (wt%) of IOT and BFS.

Components (%)	SiO_2_	Fe_2_O_3_	K_2_O	MgO	Na_2_O	Al_2_O_3_	CaO	TiO_2_
IOT	58.55	13.89	1.74	3.25	3.09	12.82	4.24	1.238
BFS	30.57	0.33	0.37	1.305	0.50	15.49	38.55	1.60

**Table 2:** Synthesis regime of IOT-BFS-based geopolymer.

Sample	IOT	Alkali Content (%)	Liquid/Solid	Alkali Activator Modulus
1#–6#	20–70%	40%	0.35	1.4
7#–14#	40%	25–60%	0.35	1.4
15#–21#	40%	40%	0.25–0.55	1.4
22#–28#	40%	40%	0.35	0.8–2

## Data Availability

All data and materials are available on request from the corresponding author.
